# Prompt Parental Reimplantation of an Avulsed Permanent Incisor in an Eight-Year-Old Child With Five-Year Follow-Up: A Case Report

**DOI:** 10.7759/cureus.105530

**Published:** 2026-03-19

**Authors:** Dhanraj Kalaivanan, Vishnu Rekha Chamarthi, Santham Krishnamoorthy, Farhana Asan, Sumaiyya Saleem, Santhosh Priya Appiya Krishnan Ramnath Babu

**Affiliations:** 1 Pediatric Dentistry, Sathyabama Dental College and Hospital, Chennai, IND

**Keywords:** case reports, child, pediatric dental trauma, tooth avulsion, traumatology

## Abstract

This report presents a successful long-term outcome of an immediate replantation of an avulsed immature permanent maxillary central incisor in an eight-year-old girl. The avulsion occurred at home during recreational activity, and the tooth was replanted within 10 min by the patient’s mother, a trained allied health professional. Clinical and radiographic assessments confirmed an open apex (Cvek stage 4), and the patient was managed in accordance with the International Association of Dental Traumatology guidelines, including flexible splinting and close follow-up. Over a five-year period, the tooth remained asymptomatic and functionally integrated. In the third year, apexification was performed using mineral trioxide aggregate owing to incomplete root development. By the fifth year, the tooth demonstrated functional success without signs of resorption. This case emphasizes the critical role of immediate management, parental awareness, and adherence to clinical follow-up protocols in ensuring the favorable prognosis of avulsed immature teeth. Furthermore, this case highlights the significance of community education and preparedness among caregivers and healthcare professionals in managing dental trauma effectively.

## Introduction

Traumatic dental injuries (TDIs) are characterized by abrupt, circumstantial, unforeseen, and accidental impact to the teeth as well as to other hard and soft tissues located within and around the oral cavity, often necessitating immediate medical intervention [1]. The incidence of avulsion of permanent teeth ranges from 0.5% to 16% of all dental injuries [2]. Avulsion, also referred to as exarticulation, denotes the complete displacement of a tooth from its alveolar socket owing to traumatic forces. Several studies have established that avulsion is the most critical among various luxation injuries.

Immediate replantation is the preferred therapeutic intervention for an avulsed tooth; however, it is not always feasible [3]. Immediate replantation is often infeasible because of comorbidities such as severe injuries, complex recipient site damage, the patient’s trauma-related emotional state, and insufficient healthcare access and knowledge among both the public and dental professionals [4]. The prognosis of a replanted tooth remains uncertain, as it may require extraction at a subsequent point in time owing to potential complications such as surface resorption, replacement resorption, and inflammatory resorption. Nevertheless, the decision to refrain from replanting the avulsed tooth is irreversible; thus, every possible effort should be made to facilitate its replantation [3,5].

Dental injuries are predominantly observed in children aged seven to 12 years; therefore, caregivers, including parents, primary school educators, and school nurses, should be adequately informed regarding the management of such injuries [6]. Nurses play a crucial role in emergency preparedness and often represent the sole category of healthcare professionals equipped to address dental emergencies. Educating nurses in this domain is pragmatic, given that their emergency training positions them as potential collaborators with dental practitioners. Moreover, many dental avulsions transpire during nocturnal hours, during which most dental clinics are non-operational, making hospital emergency departments the primary site of care for such traumatic injuries [7]. Parents, guardians, and primary educators who commonly witness avulsion injuries should be adequately knowledgeable about the initial management, handling the tooth, transporting it, and seeking appropriate dental care to prevent unnecessary tooth loss. A lack of knowledge can lead to tooth loss [8]. This case report presents a successful five-year follow-up of an avulsed incisor replanted by a parent or an allied healthcare professional immediately after the avulsion, underscoring the significance of prompt action and the need for increased awareness in such situations.

## Case presentation

This report has been prepared in accordance with the Preferred Reporting Items for Case Reports in Endodontics (PRICE) 2020 guidelines to ensure comprehensive and standardized reporting. Informed consent was obtained from the parent before the initiation of the treatment. Table 1 gives the clinical timeline of the case and the treatment rendered during the follow-up in a sequential manner.

**Table 1:** Clinical timeline according to PRICE guidelines. IADT: International Association of Dental Traumatology; PRICE: Preferred Reporting Items for Case Reports in Endodontics; MTA: mineral trioxide aggregate

Time since trauma	Clinical events and management
0 min (trauma)	Patient sustained dental trauma (fall at home). Avulsion of tooth #21 occurred.
Within 10 min	Mother (trained allied health professional) promptly replanted avulsed tooth #21.
30 min	Patient presented to the pediatric and preventive dentistry department with gingival bleeding and grade 3 mobility of tooth #21. Clinical and radiographic examination revealed grade 3 mobility, sulcular bleeding, an uncomplicated crown fracture of teeth #21 and #22, an open apex of #21 (Cvek stage 4), and no alveolar fracture. Flexible composite splint placed as per IADT guidelines for 2 weeks.
2 weeks post-trauma	Splint removal performed. The patient was asymptomatic. Esthetic composite restorations were completed for teeth #21 and #22 (fractured fragment unavailable).
4 weeks post-trauma	Radiographs showed normal periapical tissues, satisfactory healing with early mesial surface changes on tooth #21.
6 months post-trauma	Follow-up radiographs showed no periapical pathology, persistent open apex, and stable mesial surface changes.
12 months post-trauma	Clinical and radiographic evaluations confirmed that the tooth was asymptomatic, with no signs of resorption, and an open apex persisted.
24 months post-trauma	Continued asymptomatic status; radiographs showed no progressive resorption and a persistent open apex.
3 years post-trauma	Patient presented with pain in tooth #21. Radiographs confirmed an immature apex (Cvek stage 4). Management included apexification using the MTA apical barrier technique (single visit), followed by conventional obturation with gutta-percha at the next visit.
5 years post-trauma (after 2-year lapse)	Patient returned for routine evaluation, which showed tooth #21 to be clinically and radiographically asymptomatic, functional retention of replanted tooth #21, and apical closure of tooth #22.

An eight-year-old girl presented along with her mother for urgent care at the Department of Pediatric and Preventive Dentistry, Sathyabama Dental College and Hospital, Chennai, exhibiting symptoms of gingival hemorrhage and a medical history of a fall sustained during recreational activities within her residence 30 min ago. Upon conducting a thorough anamnesis, the mother revealed that she had inadvertently discovered that the child’s upper left front tooth had been entirely avulsed from its alveolar socket. She had promptly repositioned the tooth back into the socket within 10 min of the trauma after cleaning the teeth with tap water. Upon further inquiry into her rationale for the replantation procedure, she articulated that she was a certified and trained professional in the allied healthcare field.

**Figure 1:**
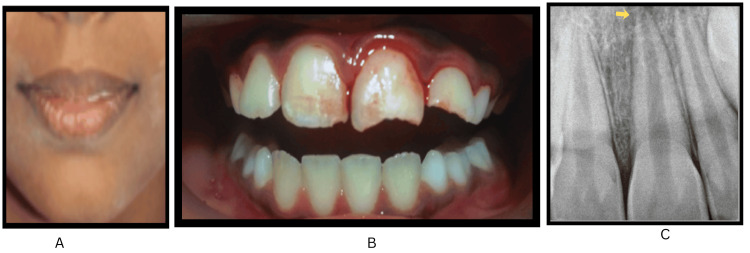
Clinical and radiographic images of the replanted maxillary left central incisor with associated crown fractures. (A and B) Intraoral and extraoral clinical photograph showing the replanted avulsed maxillary left central incisor (tooth #21) with associated uncomplicated crown fractures in teeth #11, 21, and 22. (C) Immediate intraoral periapical radiograph confirming the proper positioning of the replanted tooth #21, which presents with an open apex consistent with Cvek stage 4 root development (marked by arrow).

On clinical assessment, she had a convex facial profile, and the replanted upper left central incisor (tooth no. 21) exhibited grade 3 mobility and sulcular bleeding along with an uncomplicated crown fracture. Furthermore, an uncomplicated crown fracture was observed in tooth no. 22 (Figure 1, panels A and B).

**Figure 2:**
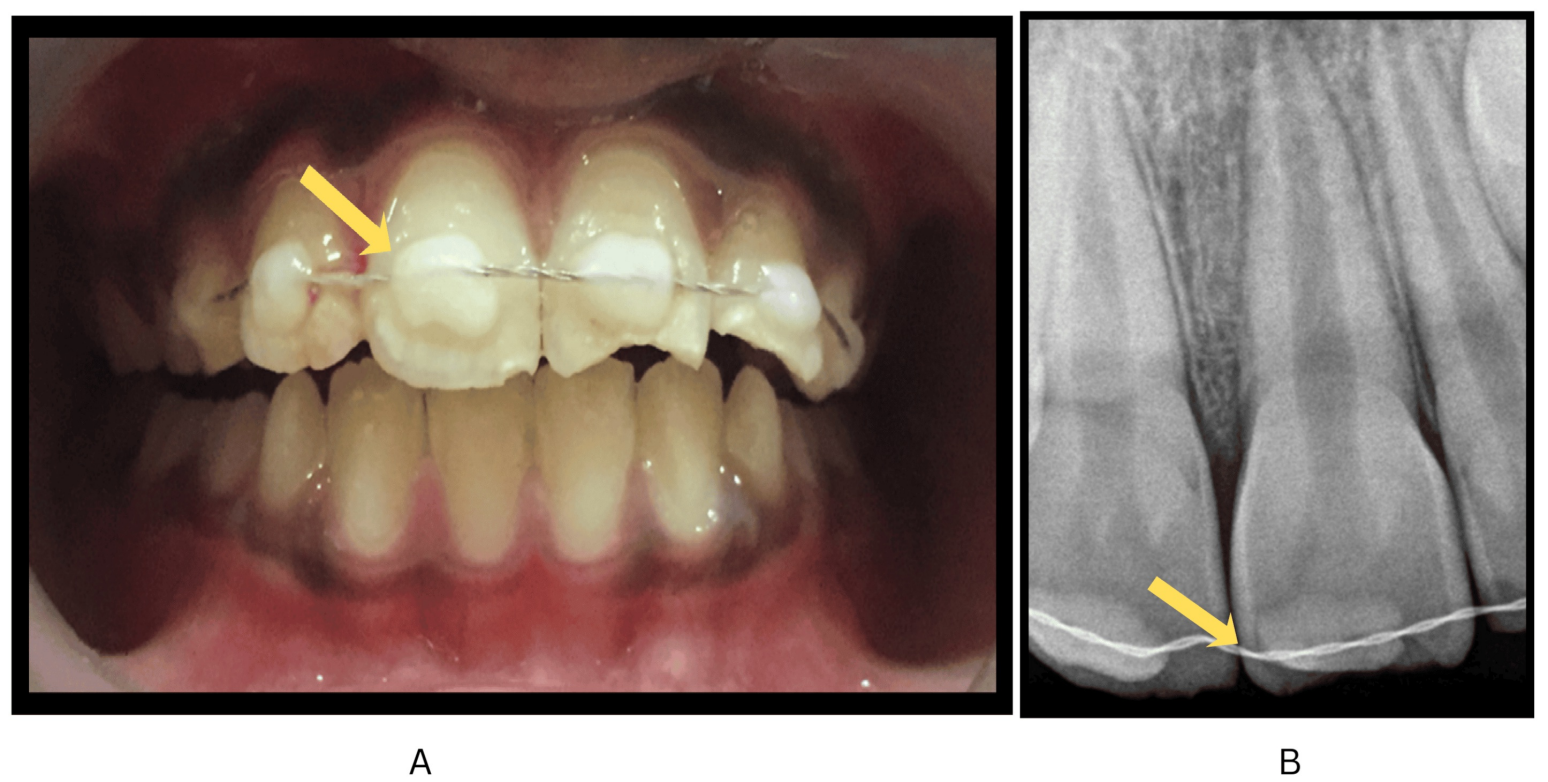
Clinical photograph and periapical radiograph demonstrating flexible splinting of teeth #11, 21, 12, and 22. (A) Intraoral clinical photograph showing flexible composite splinting involving teeth #11, 21, 12, and 22. (B) Intraoral periapical radiograph demonstrating the same splinting configuration in relation to teeth #11, 21, 12, and 22 (marked by arrows).

Parental guidance was sought to locate the tooth fragment at home for potential reattachment during the subsequent visit. The clinical position of replanted tooth no. 21 was evaluated, followed by saline irrigation to facilitate debridement and exclude associated lacerations. Radiographic analysis confirmed the integrity of the replanted tooth, showing an open apex (Cvek stage 4) and the absence of dentoalveolar fractures (Figure 1, panel C). Following International Association of Dental Traumatology (IADT) guidelines, a flexible composite splint was applied for two weeks (Figure 2, panels A and B).

**Figure 3:**
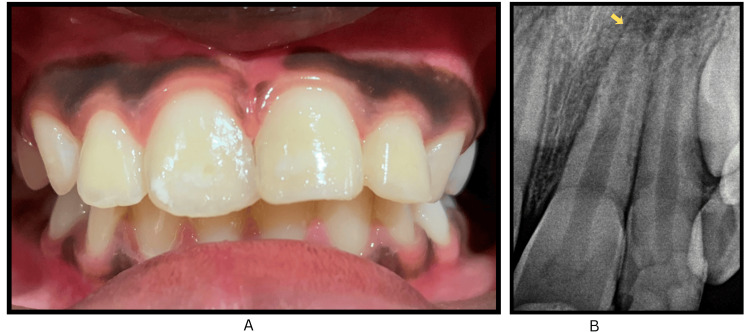
Clinical photograph and periapical radiograph after splint removal showing composite restorations and open apex of the replanted incisor. (A) Intraoral clinical photograph showing composite restorations completed in teeth #11, 21, and 22, following the removal of the flexible splint after four weeks. (B) Intraoral periapical radiograph taken at four weeks demonstrating the open apex of tooth #21, consistent with Cvek stage 4 root development (marked by arrow).

The patient remained asymptomatic after 14 days; therefore, the planned splint removal was performed, and radiographs were obtained. Esthetic restorations were performed on the upper central and lateral incisors owing to the unavailability of the fractured incisal fragment (Figure 3, panels A and B).

**Figure 4:**
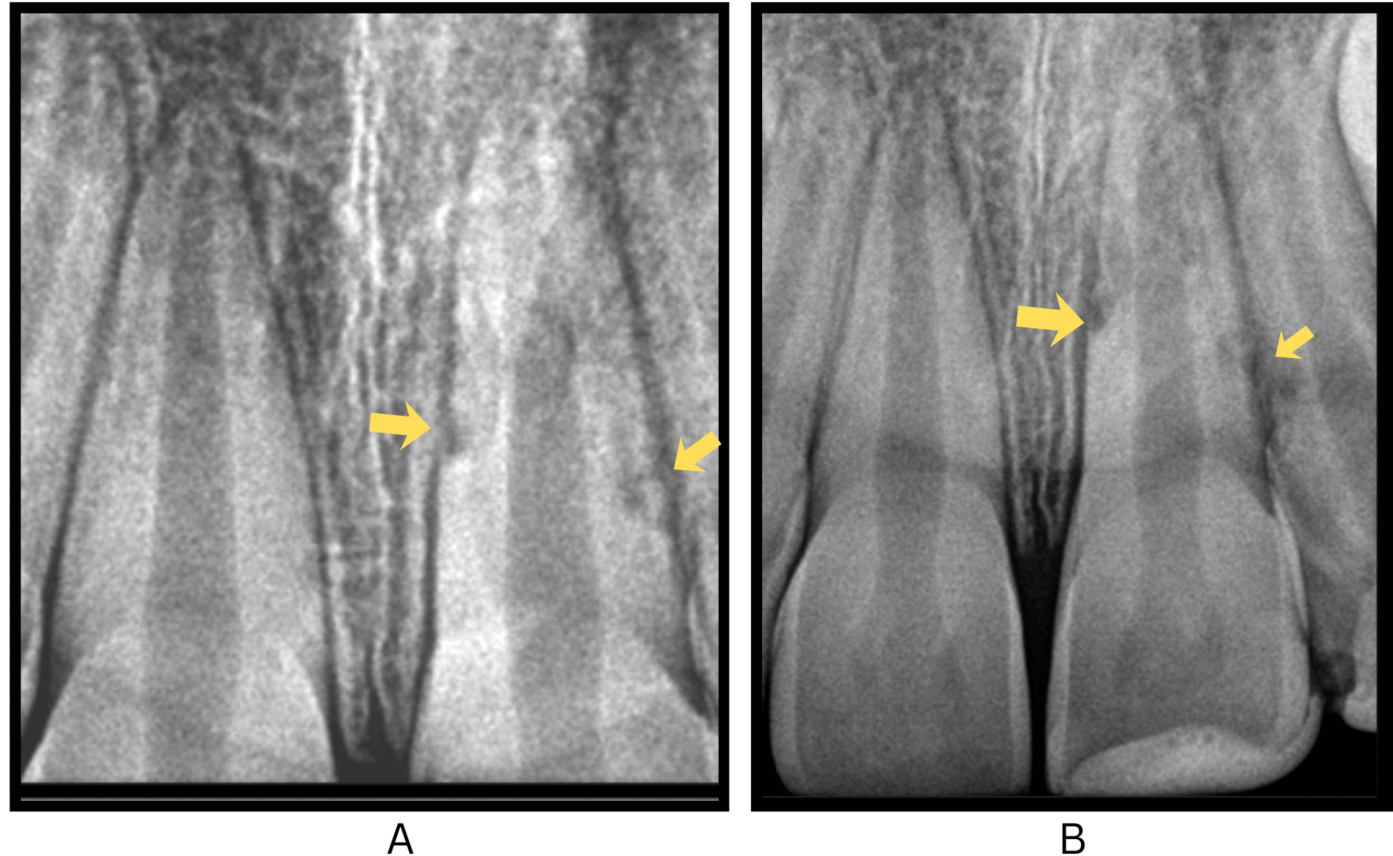
Follow-up periapical radiographs showing localized external root resorption in the replanted maxillary left central incisor. (A) Intraoral periapical radiograph taken four weeks post-replantation showing early changes of resorption marked by arrows on the mesial and distal surface of tooth #21, with the apex remaining open. (B) Intraoral periapical radiograph taken six months post-replantation showing localized resorptive changes on the mesial and distal surface of tooth #21 (marked by arrows), which appear stable and non-progressive. The apex remains immature.

**Figure 5:**
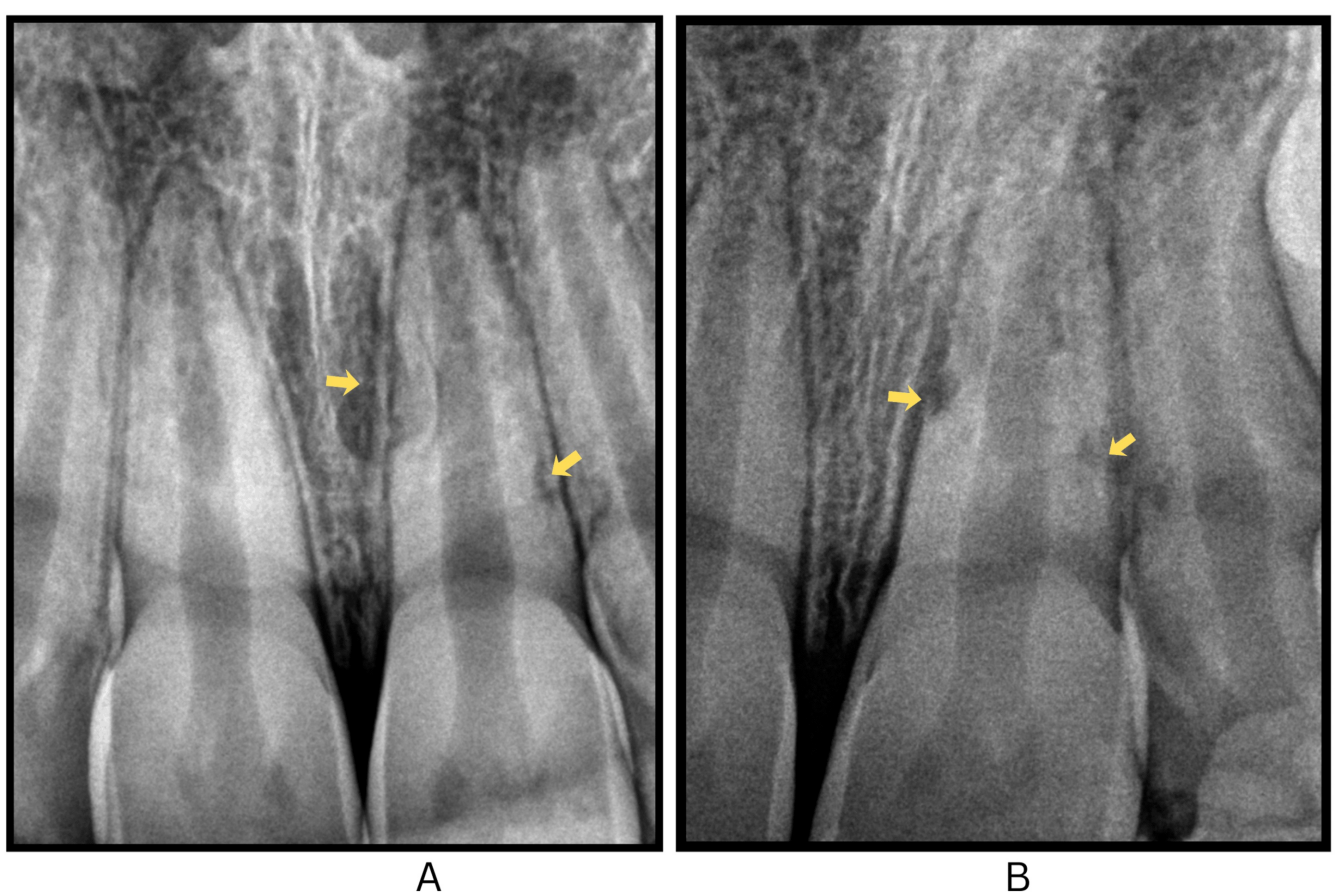
Long-term follow-up periapical radiographs of the replanted maxillary left central incisor. (A) Intraoral periapical radiograph taken one year post-replantation showing stable, non-progressive resorptive changes on the mesial and distal surface (marked by arrows) of tooth #21, with the apex remaining immature. (B) Two-year follow-up radiograph demonstrating tooth #21 with a persistent open apex.

Radiographs taken after four weeks indicated normal periapical tissues and satisfactory healing, with slight resorptive changes observed on the mesial and distal surfaces of tooth 21 (Figure 4, panel A). Six-month follow-up radiographs signified the absence of periapical alterations, showing an incomplete open apex, with the mesial and distal surface resorptive changes remaining stable (Figure 4, panel B). During subsequent follow-ups at 12 and 24 months, the patient remained asymptomatic, with vitality tests showing a delayed response in tooth 21 and radiographs demonstrating an open apex and non-progressive resorptive changes (Figure 5, panels A and B).

**Figure 6:**
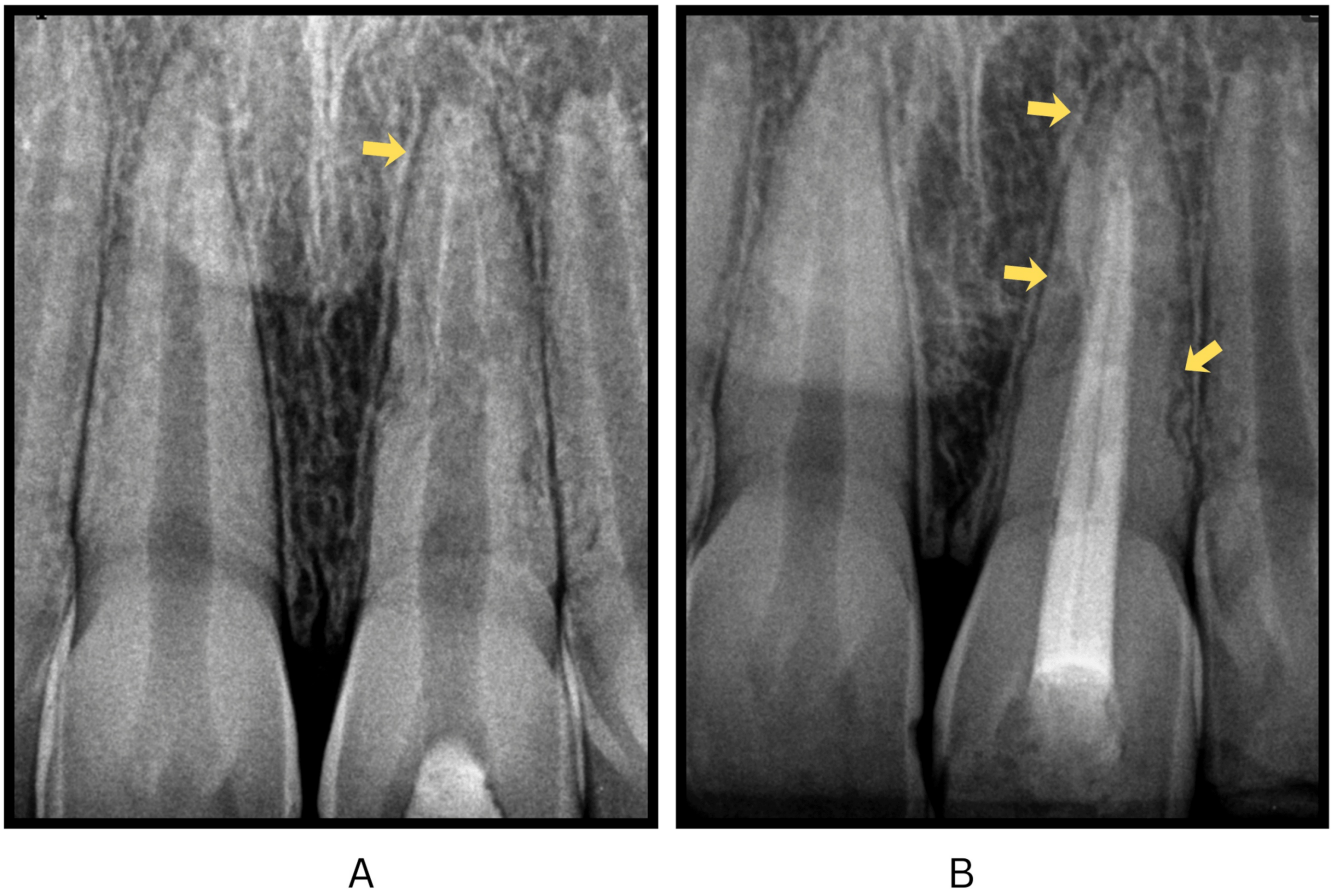
Periapical radiographs demonstrating MTA apexification and subsequent obturation of the replanted maxillary left central incisor. (A) Mineral trioxide aggregate (MTA) apexification (marked by arrow) performed during the three-year follow-up using the apical barrier technique in #21. (B) Intraoral periapical radiograph following obturation of tooth #21, showing successful apical closure and evidence of satisfactory periapical healing (marked by arrows).

In the third year, the patient presented with pain in the replanted tooth, and radiographs confirmed an immature apex (Cvek stage 4), prompting apexification using the mineral trioxide aggregate (MTA Angelus) apical barrier technique in a single visit. The next appointment involved conventional obturation with gutta-percha, confirming apical patency (Figure 6, panels A and B).

**Figure 7:**
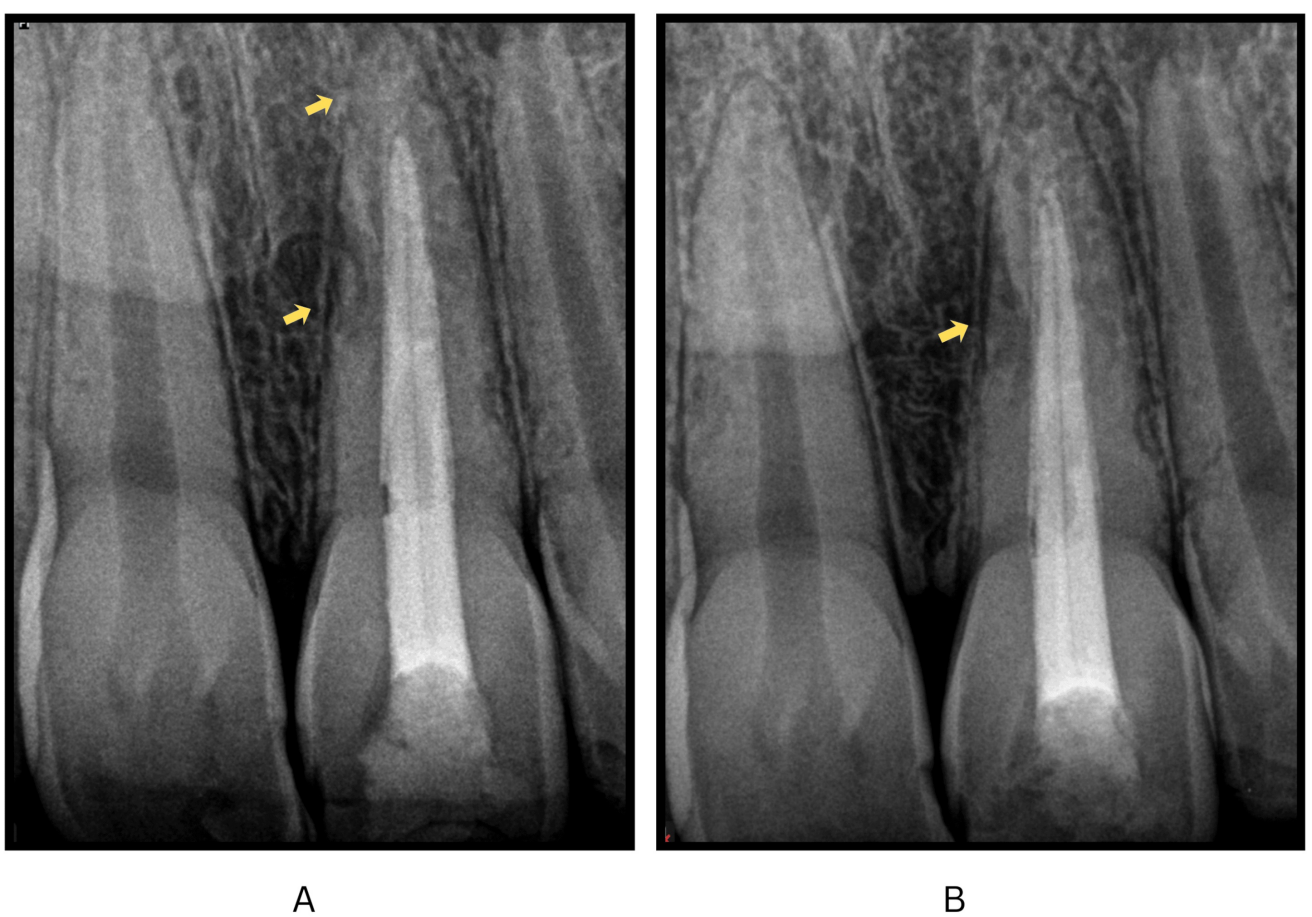
Long-term follow-up periapical radiographs demonstrating clinical stability of the replanted maxillary left central incisor. (A) Four-year follow-up radiograph showing an asymptomatic tooth #21. The arrows indicate the resolution of the lesion. (B) Five-year follow-up radiograph showing an asymptomatic tooth #21, with arrows indicating resolution of the lesion.

**Figure 8:**
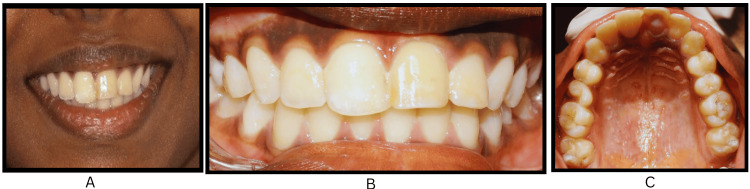
Extraoral and intraoral photographs at five-year follow-up showing the replanted maxillary left central incisor. (A-C) Extraoral and intraoral photographs of the patient at five-year follow-up showing a favorable esthetic and functional outcome of promptly replanted tooth #21.

After a two-year absence from follow-up, the patient returned for a routine examination, revealing that the replanted tooth was clinically and radiographically asymptomatic (Figure 7, panels A and B). After five years of follow-up, clinical and radiographic evaluations established the successful functionality of tooth 21 and apical closure of tooth 22 (Figure 8, panels A-C).

## Discussion

Traumatic dental injuries (TDIs) are one of the most widespread non-communicable diseases [6]. These injuries exhibit a pronounced predilection for two specific age demographics, which are also incidence peaks (i.e., two to four years of age and nine to 10 years of age for both genders). The prevalence of TDIs in the primary dentition has been documented to be 22.7%, whereas that in the permanent dentition has been reported to be 15.2% [6]. This finding highlights the fact that most individuals sustain injuries before reaching the age of 18 years. During this period, they typically reside with their parents or remain dependent on them, which is aligned with our case. Several scholars have also emphasized that the home environment constitutes one of the most common sites of injury among children, as observed in the present case [9,10].

Dental avulsion comprises approximately 0.5-16% of TDIs. The prevalence of dental avulsions following traumatic incidents ranges from 7% to 13% in primary dentition [3]. This finding suggests that the inherent properties of the pliable alveolar bone and the delicately organized periodontal ligament may render individuals more susceptible to avulsion [3]. Consequently, educational resources regarding the fundamentals of prevention and emergency management of TDIs should be provided to parents [11]. This initiative serves a dual function; first, it equips parents to manage the emergency circumstances encountered by their child adeptly and with composure, and second, it enhances their understanding of the significance of comprehensive dental interventions and follow-up care to mitigate the risk of adverse sequelae associated with TDIs [9]. In the present case, the parent (specifically, the mother), being an allied healthcare professional, attempted to replant the avulsed tooth (#21) into its socket and reported to us within the critical time frame. Given that this tooth was a young permanent tooth classified as having an open apex (Cvek stage 4) and was replanted promptly within the critical period, it was an optimal case for revascularization. In our case, in accordance with the International Association of Dental Traumatology (IADT) recommendations, extensive efforts were made to preserve the tooth's vitality owing to its immature apex. A semirigid splinting technique was used, which allows physiological movement of the tooth during healing and remains in place for a minimal period of 14 days as per IADT guidelines, to minimize the occurrence of ankylosis [3]. Furthermore, the patient was reviewed after 24 h, two weeks, four weeks, six months, and annually for five years according to IADT guidelines. The replanted tooth was clinically and radiographically asymptomatic after conventional obturation at the third year of follow-up, with a mineral trioxide aggregate (MTA) apical barrier technique.

The success rate of tooth replantation fluctuates between a low of 4% and a high of 50%, while the occurrence of replacement resorption in cases of avulsion has been reported to be 55% [12]. A clinical investigation with an extended follow-up period noted that the immediate replantation of immature teeth is associated with a lower risk of ankylosis than mature teeth [13]. Müller et al. stated that tooth avulsion constitutes a significant dental trauma characterized by an unpredictable prognosis [14]. According to the authors, functional healing was observed in 26.5% of avulsion instances and 51.0% of replanted teeth impacted by replacement resorption and ankylosis, amounting to a loss rate of 24%. The predominant cause of tooth loss was replacement resorption, followed by inflammatory resorption [14]. A recent retrospective study showed that the five-year survival rate of replanted avulsed teeth was 47.5%, with immature teeth surviving better than mature teeth, which agrees with our present case [15]. In favorable replantation cases, the resorptive defect appears only in small, demarcated areas for a limited period, ensuring an acceptable prognosis even if ankylosis and/or infra-position occur; this condition is termed “functional healing” [16]. Such healing was detected in our case in the first and second years but was not evident at the five-year follow-up. However, delay in pulpal treatment in the replanted teeth could be a drawback, which could be one of the etiologies of the resorptive changes noted in the follow-up period. However, the patient was asymptomatic in the two years of follow-up. Also, the parent handling of the tooth was unclear, and the cleaning of the surface by tap water could also lead to the resorptive changes. These findings are the results of our long-term follow-up for five years, which adds to the strength of our study. However, the diagnosis of avulsion was solely based on the clinical examination and the patient’s reporting history, which could be a limitation of this study.

## Conclusions

In such replantation cases, parents’ role and knowledge are essential for the prognosis and success of the replantation. In this instance, the mother’s knowledge and her prompt response in the form of replantation in the socket immediately after the avulsion played a significant role in the successful tooth replantation. This observation underscores the need for increased awareness. Furthermore, the prolonged clinical monitoring substantiated the functional efficacy of the replanted tooth and its complications. This emphasizes the critical significance of extended follow-up for such replanted teeth. However, these findings are derived from a single case report, which needs to be taken cautiously. Moreover, education campaigns on first aid for dental trauma should be conducted across the globe for allied healthcare professionals and the general public to provide broader implications in the prevention and emergency management of injured teeth. Such measures can enhance the prognosis of avulsed teeth and restore the healthy and happy smiles of children.
